# Effect of Marital Status on Women’s Quality of Life: A Cross-Sectional Study of Urban Women in Chengalpattu District, Tamil Nadu

**DOI:** 10.7759/cureus.68867

**Published:** 2024-09-07

**Authors:** Revathy DR, Roshni Mary Peter, Logaraj M, Anantharaman VV

**Affiliations:** 1 Community Medicine, Sri Ramaswamy Memorial (SRM) Medical College Hospital and Research Centre, SRM Institute of Science and Technology, Chengalpattu, IND

**Keywords:** married, mental health, physical health, quality of life, social health, unmarried

## Abstract

Background

Quality of life (QoL), as defined by the World Health Organization, is a subjective evaluation influenced by cultural and environmental factors. It is a complex, multidimensional concept that encompasses various aspects of an individual's well-being. This study aimed to understand how marital status affects the QoL of women by comparing married and single women residing in the urban field practice area of a tertiary care Hospital.

Methods

This cross-sectional study includes 200 women, comprising 100 single and 100 married participants, aged 20-60 years. Data were collected using the WHO-QOL-BREF questionnaire, which assesses the following four domains of QoL: physical, psychological, social, and environmental. Demographic information was also gathered. Statistical analysis was performed using SPSS Version 25.0.

Results

Significant differences were found between single and married women in age distribution (p<0.0001), family type (p=0.002), presence of children (p=0.001), educational qualifications (p=0.02), and family income (p<0.0001). Married women reported higher median family incomes and better QoL scores in the social domain (p<0.0001), while single women were predominantly younger and more likely to live in nuclear families. No significant differences were observed in the physical, psychological, or environmental domains of QoL between the groups.

Conclusion

The study highlights the impact of marital status on various QoL aspects, with married women experiencing better social support and personal relationships. Despite this, the absence of significant disparities in other QoL domains suggests that marital status does not uniformly affect overall QoL.

## Introduction

Quality of life (QoL) is defined as individuals' perceptions of their position in life, considering the culture, value systems, goals, expectations, standards, and concerns relevant to them, as outlined by the World Health Organization in 1996. This definition emphasizes that QoL is a subjective evaluation shaped by cultural, social, and environmental settings. Since QoL is based on personal perception, it does not lend itself to objective measurement [1-3].

QoL is a complex concept that encompasses physical, psychological, social, and environmental domains, all of which contribute to an individual's overall well-being. QOL is a multidimensional rather than unidirectional concept [4]. Understanding how these domains are interrelated is vital for evaluating the impact of different life conditions such as marital status on QoL. Marital status can significantly affect life experiences. Single and married women encounter different social, emotional, and practical circumstances that may affect their QoL in various ways [5,6].

Women's QoL is a crucial issue as they often face various challenges and constraints in both their personal and professional lives [7]. It is essential to recognize and address the factors that impact women's QoL regardless of their marital status. The QoL of married and unmarried women significantly affects their overall well-being and happiness [8]. While there has been extensive research on the QoL of groups such as the elderly, children, individuals with disabilities, and those suffering from diseases such as cancer, relatively little attention has been paid to women's QoL, particularly regarding their marital status [5,9].

This study aimed to explore these relationships by examining QoL across these domains among single and married urban women, providing an understanding of how marital status influences various aspects of life and contributing to a better understanding of the factors affecting the well-being of urban women.

## Materials and methods

Study design and setting

This cross-sectional study was conducted in the urban field practice area of a tertiary care hospital. The study population consisted of women residing in this area, categorized into single (unmarried, divorced, separated, or widowed) and married.

Sampling technique

A probability proportional sampling was employed to select both single and married women. Maraimalai Nagar, a municipal city located in Chengalpattu taluk of Kancheepuram district, comprises of 21 wards. According to the 2011 Population Census [10], Maraimalai Nagar had 19,970 families and a total population of 81,872, with 41,920 males and 39,952 females, resulting in an average sex ratio of 953 females per 1,000 males. The wards covered by the SRM Urban Health Training Centre include wards 4, 5, 6, 7, and 8.

Sample size

Based on a study conducted in Haryana, where the mean scores of overall QoL among married and non-married women were 82.6±6.86 and 79.6±5.28, respectively, the minimum sample size required was calculated to be 32 in each group at a 95% confidence interval. To ensure robustness and representativeness, we included 100 single women and 100 married women, making a total sample size of 200 [5].

\[ n = \frac{\left( Z_{\alpha/2} + Z_{1-\beta} \right)^2 \left( \sigma_1^2 + \sigma_2^2 \right)}{(\mu_1 - \mu_2)^2} \]

Inclusion criteria

Women aged 20-60 years, residing in an urban field practice area for at least one year, and willing to participate in the study were included.

Exclusion criteria

Women with severe mental illness or cognitive impairment, which could affect their ability to respond accurately to the questionnaire, were excluded.

Data collection

Data were collected using a structured questionnaire that included demographic information and the World Health Organization QoL (WHO-QOL-BREF) instrument from March 2023 to March 2024. The WHO-QOL-BREF assesses the following four domains of QoL: physical health, psychological health, social relationships, and the environment. A survey method was used for data collection, with the participants providing clear instructions on how to complete the questionnaire.

The demographic information collected included age, religion, type of family (nuclear, joint, three-generation, single), number of children, work status (working or non-working), educational qualification, and family income. Additionally, QoL was assessed across physical, psychological, social, and environmental domains.

Statistical analysis

The data were entered into Microsoft Excel and analyzed using SPSS Version 25.0 (IBM Corp., Armonk, NY). Descriptive statistics, such as frequencies and percentages, were used to summarize categorical variables. Means and standard deviations were calculated for the continuous variables. The chi-square test was used for categorical data, independent t-tests were used to compare the mean scores between the two groups, and the Mann-Whitney U test was used for family income. Statistical significance was set at p < 0.05.

## Results

The study included 200 women, divided equally between single (100) and married participants (100). The majority of participants were in the age group of 20-29 years (n=70), while the age group of 40-49 years was the least represented (n=31). Most participants identified as Hindu (n=154) and lived in nuclear families (n=124), followed by joint, three-generation, and single households. Additionally, the majority had children (n=163) and were employed (n=117).

Since all participants were divided into categorical variables, we used the chi-square test for analysis. Among the single women, 50% were aged 20-29, while 28% of married women were aged 50-60, showing a significant difference in age distribution (p < 0.0001). Although Hindu participants were predominant in the study, religion did not show a significant association with marital status. In terms of family type, a majority belonged to nuclear families, with 66% of single and 58% of married women living in such households, showing a significant difference (p = 0.002). The presence of children and educational level also showed significant differences, while work status did not have a significant association with marital status (Table 1).

**Table 1:** Demographic and socioeconomic characteristics by marital status

Variable		Marital status		P-value
		Single	Married	
Age group, years	20-29	50 (50%)	20 (20%)	<0.0001
	30-39	15 (15%)	33 (33%)	
	40-49	12 (12%)	19 (19%)	
	50-60	23 (23%)	28 (28%)	
Religion	Hindu	76 (76%)	78 (78%)	0.512
	Christian	19 (19%)	20 (20%)	
	Muslim	5 (5%)	2 (2%)	
Type of family	Nuclear family	66 (66%)	58 (58%)	0.002
	Joint family	11 (11%)	24 (24%)	
	Three-generation family	14 (14%)	18 (18%)	
	Single	9 (9%)	0	
Children	No	28 (28%)	9 (9%)	0.001
	Yes	72 (72%)	91 (91%)	
Work status	Working	54 (54%)	63 (63%)	0.196
	Non-working	46 (46%)	37 (37%)	
Educational qualification	Illiterate	13 (13%)	17 (17%)	0.02
	Primary school	61 (61%)	35 (35%)	
	Middle school	5 (5%)	10 (10%)	
	High school	4 (4%)	13 (13%)	
	Higher secondary	3 (3%)	6 (6%)	
	Graduate	13 (13%)	16 (16%)	
	Post-graduate	1 (1%)	2 (2%)	
	Professional	0	1 (1%)	

The median family income was analyzed using the Mann-Whitney U test to determine if there was a significant difference between single and married individuals. The results revealed a significant disparity in family income between the two groups, with single individuals having a median income of INR 15,000 and married individuals having a higher median income of INR 25,000 (p < 0.0001) (Table 2).

**Table 2:** Comparison of family income by marital status using the Mann-Whitney U test

Variable		Family Income			P-value
		Median	Percentile 25	Percentile 75	
Marital status	Single	15,000	8,000	20,000	<0.0001
	Married	25,000	16,500	40,000	

The QoL scores for single and married women were compared across four domains: physical, psychological, social, and environmental. Independent t-tests were used to assess the differences between the two groups. Except for the social domain, where a significant difference was found (p < 0.0001), no other domains showed significant differences (Table 3).

**Table 3:** Comparison of quality-of-life domains by marital status

Variable	Marital Status		P-value
	Single	Married	
Physical domain	58.52 ±15.79	61.41 ±13.09	0.227
Psychological domain	60.52 ±15.69	63.45 ±16.06	0.293
Social domain	55.26 ± 21.11	72.95 ±21.26	<0.0001
Environment domain	63.81 ±18.15	68.31± 15.78	0.113

## Discussion

The study provides a detailed examination of QoL across multiple domains, offering a nuanced understanding of the differences between single and married women. By including women from various age groups and family structures, the study allows for a broad exploration of factors influencing QoL. Comprising 100 married and 100 single women, the study aimed to compare the QoL of these groups, providing valuable insights into their demographic, socioeconomic, and QoL differences. The age distribution showed significant variation, with single women predominantly younger (20-29 years) compared to married women, who were more evenly distributed across age groups. This disparity might reflect societal norms and cultural expectations regarding marriage, particularly in urban settings, where educational and career pursuits may delay marriage. Additionally, religious distribution did not differ significantly between groups, indicating that marital status was not influenced by religious affiliation in this cohort. However, family structure showed notable differences: single women were more likely to live in nuclear families, while married women were more often part of joint or three-generation families.

Verma et al. found that around half of married (45%) and unmarried (55%) women were between 26 and 30 years of age. In terms of education, most women in both groups had education up to post-matric and graduation levels, with a smaller proportion being postgraduates. Around half of the married women were homemakers, while more than half of the unmarried women were students [5].

Moreover, we noted that the presence of children was significantly higher among married women, which was expected given the traditional association between marriage and childbearing. Family income disparities were also evident, with married women reporting higher median income. Verma et al. found that a large proportion of both married and unmarried women belong to small or large families. Around 65% of married women and 70% of unmarried women were from joint families. Around half of the respondents were from families with a monthly income of Rs.50,000-100,000 [5,11,12].

Furthermore, we evaluated QoL across the physical, psychological, social, and environmental domains. No significant differences were found between the physical and psychological aspects of single and married women. However, married women reported notably higher scores in the social domain, reflecting better personal relationships and social support. Although the differences in the environmental domain were not statistically significant, married women had higher scores, possibly because of the benefits of a stable, dual-income household, and supportive family.

Kaur et al., Han et al., Bhatia et al., and Pereira et al. found that married women had better QoL compared to single women and those experiencing marital problems such as divorce or separation. Similarly, married men scored higher in QoL than those who were single or had marital issues [6,13-15].

Bhatia et al. conducted a study on the QoL among married and unmarried individuals at Panjab University, Chandigarh. They found that married employees and students had higher overall QoL across all domains (physical, psychological, social, and environmental) than their unmarried counterparts did. The better QoL of married individuals was attributed to enhanced physical and psychological support and social security, while unmarried individuals experienced poorer QoL due to issues such as poor physical health, depression, social insecurities, stress, and familial and peer pressure. The study also noted that QoL decreased with increasing age, duration of marriage, and number of children [6].

A report by Pereira et al. suggests that women who maintain a distant relationship with their husbands often experience somatic symptoms, which subsequently decrease their overall QoL [15]. A study conducted by Gull et al. revealed that media use significantly negatively impacts the QoL and marital satisfaction of married couples. It causes conflicts, a lack of trust, loneliness, and negative feelings due to inappropriate posts [11]. Additionally, other studies have shown that QoL decreases with increasing age, duration of marriage, and number of children [15,16]. Moreover, QoL was found to be better in homemakers than in working or non-working individuals, as reported by Bhatia et al. [6].

Verma et al. found a significant difference in overall QoL between married and unmarried women, with married women reporting a better overall QoL. While unmarried women scored higher in physical and mental aspects, married women had better social and spiritual health. Additionally, increased smartphone use and screen time were negatively correlated with QoL, as was larger family size [5,17]. However, a study conducted by Solhi et al. and Rehman et al. found a significant correlation between physical health, education, and marital status. They discovered that women with higher education had better physical health, while married women had worse physical health [18,19].

Qadri et al. found that married individuals in District Ambala, Haryana, had a QoL score of 69.46, compared to 68.67 for unmarried individuals [20]. Similarly, Saxena et al. reported that unmarried individuals had a higher overall QoL score of 68.63 compared to 65.59 for married individuals [21]. Jain and Kumar found no significant difference in the QoL between married and unmarried adults. However, the studies revealed that life satisfaction was higher among married adults compared to their unmarried counterparts [22,23].

Future research should explore longitudinal designs to establish causal relationships between marital status and QoL. Additionally, studies should include diverse populations across different cultural and socioeconomic backgrounds to enhance generalizability.

Limitations of the study

This study had some limitations, including its cross-sectional design, which limited the ability to establish causality between marital status and QoL. The study was confined to an urban setting, and the findings may not be generalizable to rural populations or other cultural contexts. Self-reported data are subject to social desirability and recall biases. This study did not account for other variables that might influence QoL, such as employment status, health conditions, or social networks outside the family.

## Conclusions

This study provides valuable insights into the demographic, socioeconomic, and QoL differences between single and married women. Our findings highlight significant differences in age distribution, family type, presence of children, educational qualifications, and family income between the two groups. Married women were found to have higher median family incomes and significantly better QoL scores in the social domain, suggesting they benefit from stronger social support and personal relationships. In contrast, single women were more likely to be younger, live in nuclear families, and have a lower median family income. Despite these differences, no significant disparities were observed in the physical, psychological, or environmental domains of QoL.
